# Role of ethylene signalling in the formation of constitutive aerenchyma in primary roots of rice

**DOI:** 10.1093/aobpla/plu043

**Published:** 2014-07-24

**Authors:** Kenta Yukiyoshi, Ichirou Karahara

**Affiliations:** Department of Biology, Graduate School of Science and Engineering, University of Toyama, Toyama 930-8555, Japan

**Keywords:** Aerenchyma, 1-aminocyclopropane-1-carboxylic acid (ACC), ethylene, 1-methylcyclopropene (1-MCP), *Oryza sativa*, roots

## Abstract

Although the extent of aerenchyma (interconnected gas-filled space) formed in the cortex of the roots of some species can be promoted by submergence and ethylene, such roots also form a somewhat less extensive aerenchyma under well-aerated conditions. It has been unclear whether or not ethylene is also involved in promoting this constitutive aerenchyma. To confirm the potential of ethylene to stimulate aerenchyma development and test the possibility that gas regulates constitutive aerenchyma, a novel sandwich method was employed in rice roots. This involved germinating japonica rice (*Oryza sativa*) caryopses sandwiched between two agar slabs with or without 1-aminocyclopropane-1-carboxylic acid (ACC) at 1 µM. The roots were then grown for 4 days in the dark in the presence or absence of gaseous 1-methylcyclopropene (1-MCP), an inhibitor of ethylene action. Examination of aerenchyma development by light microscopy demonstrated a more extensive aerenchyma in cross-section on the ACC-treated side that also commenced closer to the root tip. In the presence of 1-MCP at 0.1 or 1 ppm, aerenchyma formation was inhibited in the presence or absence of ACC. 1-Methylcyclopropene also overcame ACC-inhibited root elongation. The results indicate that ethylene signalling is involved in aerenchyma development in primary roots of rice and that this may include the regulation of constitutive aerenchyma. In addition, root elongation was slowed in control roots in the presence of 1 ppm 1-MCP, supporting previous studies demonstrating that endogenous levels of ethylene stimulate root elongation.

## Abstract (precis)

Although aerenchyma formed in the roots of some species can be promoted by ethylene, such roots also form a somewhat less extensive aerenchyma under well-aerated conditions. It has been unclear whether ethylene is involved in promoting this constitutive aerenchyma. To test this possibility a novel sandwich method was employed in rice roots. A more extensive aerenchyma was formed on the ACC-treated side. 1-MCP inhibited aerenchyma formation in the presence or absence of ACC. The results indicate that ethylene signaling is involved in aerenchyma development in rice roots and that this may include the regulation of constitutive aerenchyma.

## Introduction

Understanding the physiological basis of flooding tolerance is an important component of breeding improved crop varieties with superior performance under flooded or submerged conditions (Ismail *et al.* 2009, 2012). Numerous wetland species, including rice, develop aerenchyma in their roots as an adaptation to low-oxygen conditions in the soil (Justin and Armstrong 1987). Aerenchyma formation in roots of crop plants is considered crucial for roots to function in oxygen-deficient soils and positive correlations have been observed between aerenchyma formation in adventitious roots and (i) shoot growth in wheat under hypoxia (Huang *et al.* 1994) or (ii) the yield of wheat cultivars grown under intermittent waterlogging conditions (Setter and Waters 2003). Not surprisingly, the importance of understanding mechanisms of aerenchyma formation in flooding tolerance has been emphasized previously for rice (Nishiuchi *et al.* 2012) and also other crop species (Yamauchi *et al.* 2013).

The induction of lysigenous aerenchyma development by flooding and other environmental factors and the underlying mechanism have been intensively investigated in maize roots. When maize roots were exposed to low-oxygen environment due to stagnant conditions, activities of 1-aminocyclopropane-1-carboxylic acid (ACC) synthase and of ACC oxidase increased (He *et al.* 1996*a*) and, as a result, enhanced ethylene production induced cell death in root cortex and induced lysigenous aerenchyma formation (He *et al.* 1996*b*). These results are supported by experiments using ethylene treatments and ethylene action inhibitors (Drew *et al.* 1979, 1981; Jackson *et al.* 1985*a*; Rajhi *et al.* 2011). Recently, Rajhi *et al.* (2011) identified genes associated with lysigenous aerenchyma formation in maize roots, supporting the mechanism of ethylene-mediated lysigenous aerenchyma formation.

Despite early indications that ethylene is not involved in aerenchyma formation in rice (Jackson *et al.* 1985*b*), Justin and Armstrong (1991), using different cultivars, showed that ethylene was capable of stimulating aerenchyma in rice roots. More recent studies have shown that aerenchyma formation is enhanced by stagnant conditions (Colmer *et al.* 2006; Shiono *et al.* 2011) and indicated that ethylene is involved in this process (Colmer *et al.* 2006; Shiono *et al.* 2008). Therefore, existence of two types of aerenchyma has been suggested in rice roots: one is inducible aerenchyma, which forms in low-oxygen environments; the other is constitutive aerenchyma, which fundamentally forms in the absence of external environmental stress as an integral part of ordinary root development (Jackson *et al.* 1985*b*). Involvement of ethylene in the formation of constitutive aerenchyma, as well as that of inducible aerenchyma (Abiko and Obara 2014), has been debated (Kong *et al.* 2009). It has been demonstrated that in *Juncus effusus* constitutive aerenchyma formation does not involve ethylene signalling (Visser and Bögemann 2006). This was also tested in rice roots using ethephon, a compound that breaks down to form ethylene (Kong *et al.* 2009). However, demonstrating that externally applied ethylene or restricted oxygen supply can stimulate aerenchyma in rice does not necessarily show that the gas regulates constitutive aerenchyma formation. Furthermore, it has been difficult to detect since the absolute amounts of aerenchyma are small and root elongation can be affected by treatments. Therefore, it remains necessary to establish the involvement of ethylene in constitutive aerenchyma formation in rice while recognizing that various external conditions can induce it.

In the previous studies, little attention has been given to aerenchyma development over time because it is difficult to determine this along the whole root (Karahara *et al.* 2004, 2008; Chen *et al.* 2011). To facilitate examination of the development of constitutive aerenchyma over time and space we have developed a unique ‘sandwich’ method, in which roots are grown between two sheets of agar media that can contain different constituents. This allows a comparison of development of internal tissues on the two opposing sides of the roots exposed to different conditions. In a previous work using this method, we were able to demonstrate that mannitol treatment promoted aerenchyma development in the primary roots of rice (Karahara *et al.* 2012).

In the present study, we used ACC as the immediate precursor of ethylene to allow ethylene enrichment principally on one side of the root. We tested whether treatment with ACC enhances aerenchyma development and also checked if constitutive aerenchyma is inhibited by 1-methylcyclopropene (1-MCP), an inhibitor of ethylene action effective at low concentrations (Sisler and Serek 1997).

## Methods

### Plant material and germination conditions

Rice (*Oryza sativa* L. ssp. *japonica* ‘Nipponbare’) caryopses were presoaked in distilled and deionized water (ddH_2_O) for 4 days at 4 °C which was changed daily. Imbibed caryopses were soaked in 70 % (w/v) ethanol for 10 s in a 2.5 % (w/v) sodium hypochlorite solution for 5 min for sterilization and then rinsed with ddH_2_O. They were then placed in a Petri dish and incubated in ddH_2_O for 24 h at 30 °C for germination.

### Treatment with ACC using a ‘sandwich’ method

**Figure 1.**
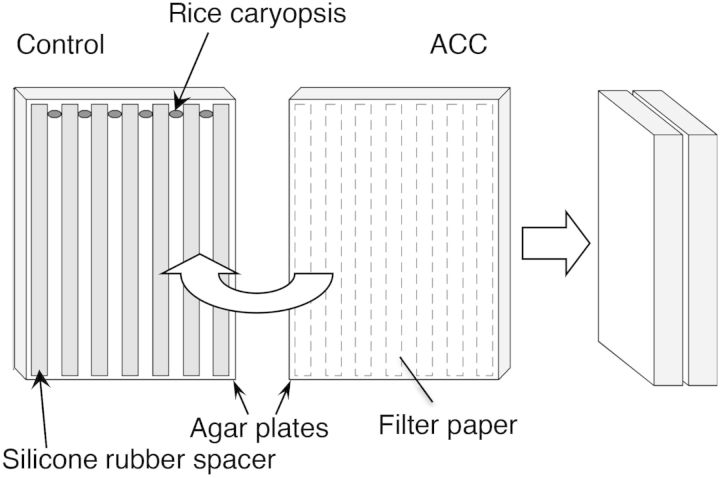
Experimental set-up of the ‘sandwich’ method used in the present study. A schematic illustration showing how rice caryopses were sandwiched between two agar slabs. Broken lines on the right-hand agar slab show spacer position. Figure modified from Karahara *et al.* (2012).

Germinating rice caryopses were sandwiched between two 2 % (w/v) agar slabs 12 mm thick containing 1/10 strength Hoagland medium (Sigma-Aldrich Japan K.K., Tokyo, Japan). The surface of each agar slab facing the rice caryopses was covered with a sheet of filter paper. Spacers 0.5 mm thick and made of silicone rubber ensured that the sheets of filter paper on each agar slab did not touch (Fig. 1). For the unilateral treatment of roots with ACC, ACC was added to one of the two agar media to a final concentration of 1 or 10 µM. The two agar slabs sandwiching the roots were placed vertically in a dark box. Roots were allowed to grow at 25 °C in the dark for 4 days, attached to both agar slabs.

To examine whether there is a difference in the elongation of rice roots between those exposed to ACC on one side or both sides, compared with controls, we compared the length of 4-day-old roots treated on one side (control/ACC, referred to as unilaterally treated roots) with those without treatment (control/control, referred to as control roots), and with those treated on both sides (ACC/ACC, referred to as bilaterally treated roots).

### Light microscopy

To distinguish between control and the ACC-treated sides in cross-section, the control side was stained with Evans blue dye or marked by making a shallow incision using a razor blade along the root axis (Karahara *et al.* 2012). When roots were stained with Evans blue, the control side of the agar slab was temporarily replaced with 2 % (w/v) agar slabs containing Evans blue dye at a concentration of 0.1 % (w/v) for 1 h.

For observations of aerenchyma, roots with average lengths were selected for each treatment. The stained roots were then embedded in molten 5 % (w/v) agar aqueous solution just before the agar began to gel. Cross-sections (100-mm-thick) were cut basipetally from the root tip to the base at 2.5- or 5-mm intervals along the entire primary root using a linear slicer (PRO7; Dosaka EM, Kyoto, Japan). These cross-sections were inspected under bright-field optics for the presence of aerenchyma. The Evans blue stain, which shows red fluorescence under UV light, was observed under a fluorescence microscope (BX-50 FLA; Olympus Corp., Tokyo, Japan) equipped with a filter assembly for excitation by UV light (U-MWBV: excitation filter, BP400–440; absorption filter, BA475; dichroic mirror, DM-455; Olympus Corp.). Bright-field or fluorescence micrographs were taken using a digital camera (Cool Snap cf; Nippon Roper KK, Tokyo, Japan) (Nakabayashi *et al.* 2006).

### Quantitative assessment of aerenchyma

Both quantitative assessment of volume of aerenchyma (spatial comparison) and assessments of aerenchyma development over time (temporal comparison) are based on data of aerenchyma area (% of cortex) examined on cross-sections prepared from different positions along a root. We had previously verified that it is possible to assess difference in aerenchyma development between the two sides, i.e. the treated and the control, at one position and thus with both sides having the same cell age. To assess the effects of the treatments on aerenchyma development, quantification of areas of cortical aerenchyma (i.e. areas devoid of intact cells) and cortex (excluding endodermis, sclerenchyma and exodermis) in cross-sectional images was performed using Openlab Darkroom software (Improvision, Coventry, UK). Digital images of cross-sections obtained by light microscopy were divided into the two opposite treated sides of the roots (e.g. the control side and the ACC-treated side). The total area of aerenchyma was calculated as the sum of the actual cross-sectional areas of lysigenous aerenchymatous space observed on each side. The percentage of the total area of aerenchyma in the cortex in each side of the root was compared between the control sides and the ACC-treated sides in cross-sections taken 50 mm from the root tip.

Total aerenchyma volume on each side of the root was estimated as follows using Igor Pro v.5 software (WaveMetrics, Inc., Lake Oswego, OR, USA). First, the areas of aerenchyma obtained were plotted against distance from the root tip and the data interpolated using binomial smoothing (seven passes). Then, the integral function of the binomial smoothing function (seven passes) of cross-sectional area of aerenchyma, i.e. cumulative aerenchyma area along the root axis measured every 5 mm, was obtained. The *Y* coordinate of the point at which *X* coordinate (the distance from root tip) corresponds to the position of the base of the root, on the curve of the cumulative aerenchyma area, was obtained. This value was multiplied by 5 mm because cross-sections were cut at 5-mm interval to estimate the total volume of aerenchyma along the root. Statistical tests were performed using JMP v.9 software (SAS Institute Inc., Cary, NC, USA).

### Assessment of aerenchyma development over time

The distance from the root tip to where aerenchyma was formed reflects how soon it starts to develop after the cells were first produced in the apical meristem (Karahara *et al.* 2004, 2008). Assessment of the effects of treatments such as unilateral ACC application on the time aerenchyma development commenced was performed by comparing this distance, as carried out in previous studies (Karahara *et al.* 2012) but with slight modification. On a cross-sectional image, each side (e.g. the control side and the ACC-treated side) was divided into eight unit sectors. For each side, the percentage out of eight unit sectors where aerenchyma was observed was plotted against the distance from the root tip. The data were interpolated using binomial smoothing (seven passes) using Igor Pro v.5 software. The distances were compared between the control side and the ACC-treated side when the percentage reached 40 % of the interpolated data.

### Treatment with 1-MCP

The gaseous ethylene action inhibitor, 1-MCP, was supplied to the whole plants at 0.1 or 1 ppm (v/v) according to previous studies (Visser and Bögemann 2006; Rajhi *et al.* 2011) but with slight modification. To achieve the concentration of 1 ppm, 19.2 mg of Smart Fresh (Rohm and Haas Company, Philadelphia, PA, USA) was mixed with 600 µL ddH_2_O in a 1.5-mL polyethylene tube. The open polyethylene tube containing the solution of 1-MCP was placed inside a 12.8-L gas-tight plastic chamber (K-Box S70; Asbel, Nara, Japan) allowing gaseous 1-MCP to perfuse the chamber. Germinating rice caryopses sandwiched between two agar slabs were placed in the chamber and the roots were grown on for 4 days.

## Results

### Effects on root elongation of unilateral treatment of roots with ACC in the presence or absence of 1-MCP

**Figure 2.**
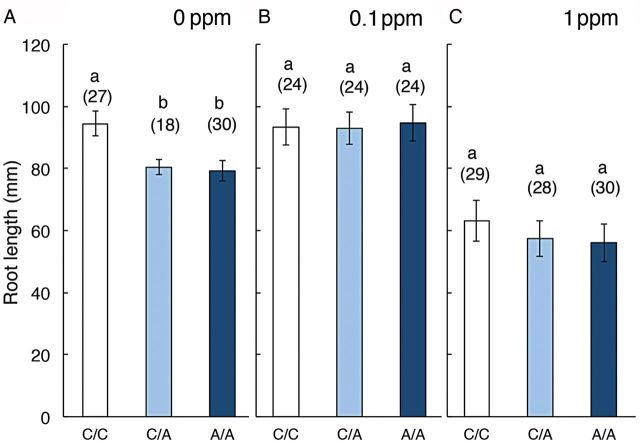
Effects of 4 days unilateral or bilateral treatment with 1 µM ACC on the length of primary rice roots in the presence or absence of 1-MCP. (A), (B) and (C) correspond to the treatment with ACC in the presence of 0, 0.1 and 1 ppm of 1-MCP, respectively. White columns, Control/Control; light blue columns, Control/ACC; dark blue columns, ACC/ACC. Different letters on the columns within each group indicate significant difference between the treatments assessed by the Tukey–Kramer HSD test (*P* < 0.05). Values are presented as the mean ± SE. The numbers of samples are shown in parentheses.

To determine the appropriate concentration of ACC for aerenchyma and growth studies, its effect on root elongation was examined at concentrations of 1 or 10 µM (data not shown). A significant inhibition of root elongation as well as a significant promotion of aerenchyma formation was observed even at 1 µM. This concentration was therefore used in subsequent experiments. The length of 4-day-old roots treated with ACC, either unilaterally or bilaterally, was significantly shorter than that of control roots (Fig. 2A). However, these significant differences in the root length treated with ACC disappeared in the presence of 1-MCP at 0.1 ppm (Fig. 2B) with no effect on roots not given ACC. At the higher 1-MCP concentration of 1 ppm (Fig. 2C), root extension was slowed in control roots and in ACC-treated roots.

### Effects of unilateral treatment of roots with ACC in the presence or absence of 1-MCP on aerenchyma formation

**Figure 3.**
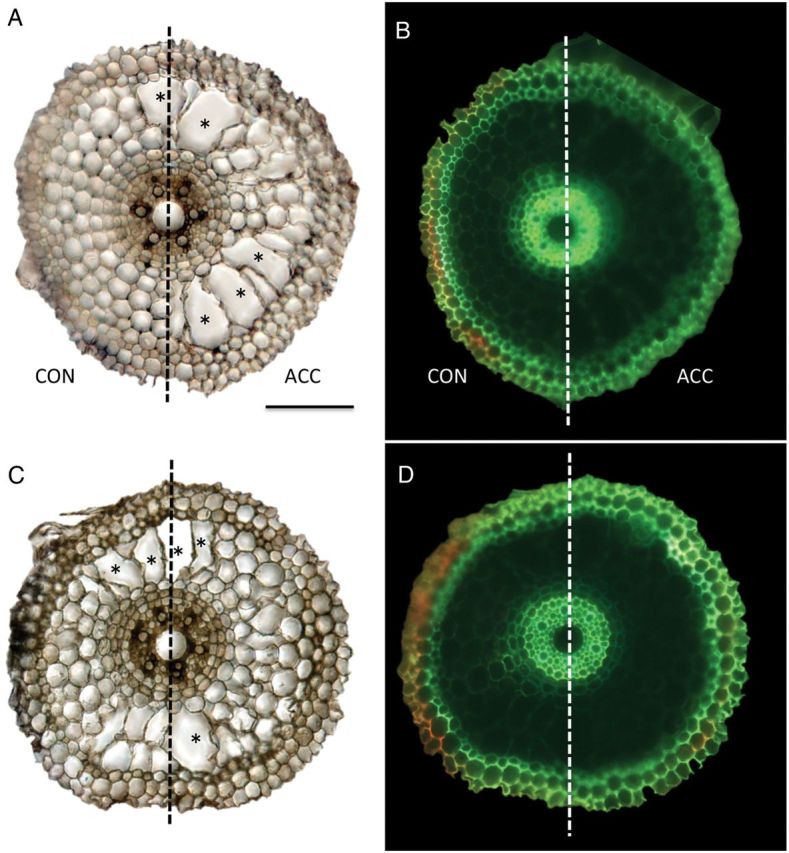
Micrographs of cross-sections of 4-day-old primary roots of rice treated unilaterally with 1 µM ACC in the absence (A, B) or presence (C, D) of 1-MCP at 0.1 ppm and stained at the root surface of the control side with Evans Blue. Bright-field views are shown in (A, C). Fluorescence views are shown in (B, D). The micrographs are of cross-sections cut at 35 mm (A, B) or 40 mm (C, D) from the root tip. Evans blue shows red fluorescence on the control side (the left side) (B, D). CON, control side; ACC, ACC-treated side. *Well-developed aerenchyma. Scale bar=100 µm.

Cross-sections were cut at 5-mm intervals along the axis of primary roots unilaterally treated with 1 µM ACC in the presence or absence of 0.1 ppm 1-MCP. In Fig. 3, the appearance of cross-sections of typical roots 35 or 40 mm from the root tip is shown. The boundary between the control side and the ACC-treated side of a cross-section was judged according to Evans blue staining (Fig. 3B and D) and the area of aerenchyma in each cortical area was measured using bright-field images (Fig. 3A and C).

**Figure 4.**
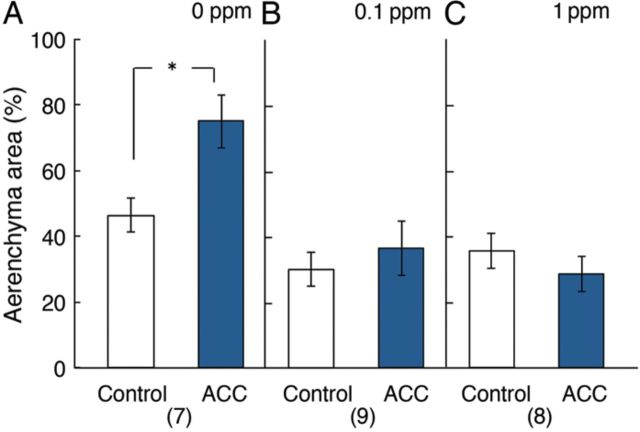
Effects of unilateral 4-day treatment of primary roots of rice with 1 µM ACC on the percentage of cross-sectional area of the cortex that comprises aerenchyma. Measurements were made 50 mm from root tip in the presence or absence of 1-MCP. (A), (B) and (C) correspond to the treatment with ACC in the presence of 0, 0.1 and 1 ppm of 1-MCP, respectively. White column, control side; blue column, 1 μM ACC treatment side. Values are presented as the mean ± SE. *P < 0.05 (Wilcoxon signed-ranks test). The numbers of samples are shown in parentheses.

When the percentage of total aerenchyma area in the cortex was compared 50 mm from root tip in roots unilaterally treated with 1 µM ACC in the presence or absence of 1-MCP at 0.1 ppm (Fig. 4), it was significantly increased from ∼45 % to over 70 % in the ACC-treated side (Fig. 4A). On the other hand, in roots unilaterally treated with ACC in the presence of 1-MCP at 0.1 or 1 ppm, the promotion of aerenchyma development in the ACC-treated side was eliminated (Fig. 4B and C).

### Effects of unilateral ACC treatment in the absence or presence of 1-MCP on aerenchyma development over time

**Figure 5.**
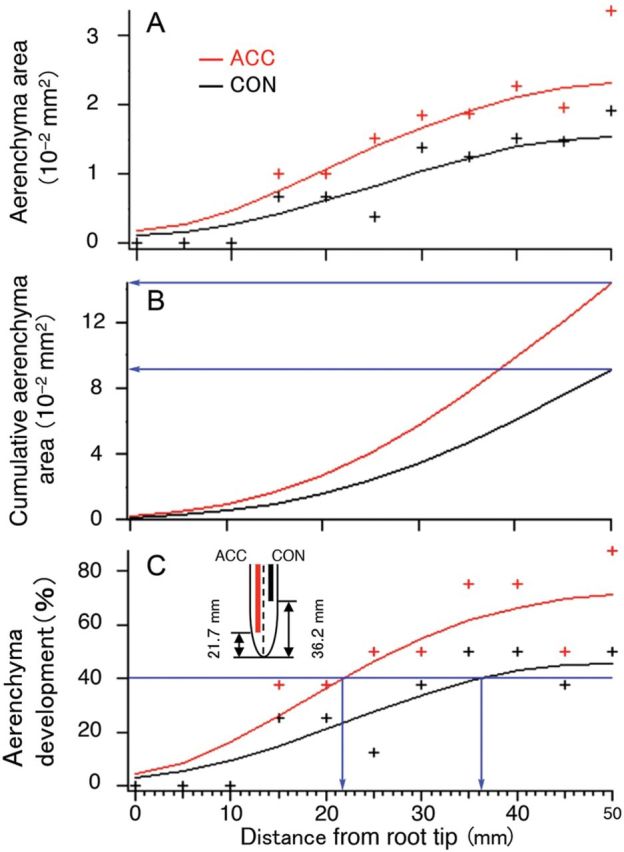
Effects of 4 days unilateral treatment of a primary root of rice with 1 μM ACC on the aerenchyma development plotted against distance from root tip. A typical 4-day-old rice root is shown. Black line, control side; red line, ACC treatment side. (A) Actual total cross-sectional area of aerenchyma in each side. The data were interpolated using bionomical smoothing (seven passes). CON, Control side; ACC, ACC-treated side. (B) Curve of cumulative cross-sectional area of aerenchyma obtained for each side as the integral function of the binomial smoothing function of cross-sectional area of aerenchyma shown in (A). (C) The percentage of total cross-sectional area of aerenchyma of the cortex in each side. The data were interpolated using bionomical smoothing (seven passes).

The effects of unilateral ACC treatment on the volume of aerenchyma were also assessed along the entire length of the roots in the presence or absence of 1-MCP at 0.1 or 1 ppm. In Fig. 5A, cross-sectional area of aerenchyma is plotted for each side against distance from the root tip and data were interpolated using bionomical smoothing. Fig. 5B shows a curve of cumulative aerenchyma area measured every 5 mm along each side of the root axis using the integral function of the binomial smoothing function of cross-sectional area of aerenchyma. Fig. 5C estimated the position along the root where 40 % of the cortex was aerenchymatous. These results indicate that ACC promoted aerenchyma along the entire treated side of the root (Fig. 5A) and that the total amount of aerenchyma along that side of the root was therefore enhanced (Fig. 5B). 1-Aminocyclopropane-1-carboxylic acid also appeared to shorten the time before aerenchyma started to form. This is indicated by the more distal position at which 40 % of the cortical area became aerenchymatous (21.7 mm rather than 36.2 mm from the tip—Fig. 5C). The value of cumulative aerenchyma area at the position of the base of the root was multiplied by 5 mm and, thereby, aerenchyma volume was estimated.

**Figure 6.**
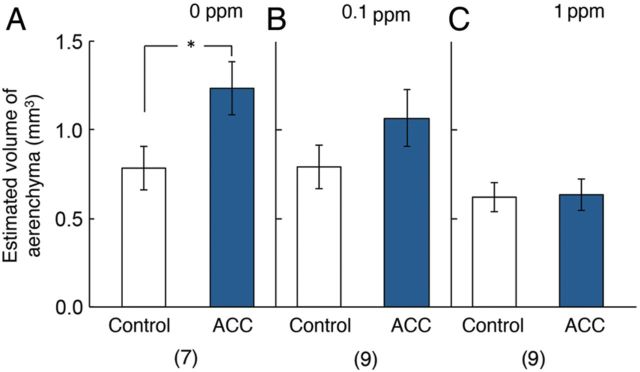
Effects of 4 days unilateral treatment of primary roots of rice with 1 µM ACC on the estimated total volume of aerenchyma along each side of the roots up to the root base in the presence and absence of 1-MCP. (A), (B) and (C) correspond to the treatment with ACC in the presence of 0, 0.1 and 1 ppm of 1-MCP, respectively. White column, control side; blue column, 1 μM ACC treatment side. Values are presented as the mean ± SE. *P < 0.05 (Wilcoxon signed-ranks test).

In a separate experiment (Fig. 6), we again showed that a root unilaterally treated with ACC in the absence of 1-MCP increased its aerenchyma on the treated side (Fig. 6A) expressed in terms of the total volume of gas space along the whole length of each side of the root. In roots unilaterally treated with ACC, applying 1-MCP at 0.1 or 1 ppm partially or completely nullified the promoting effect of ACC on aerenchyma (Fig. 6B and C).

**Figure 7.**
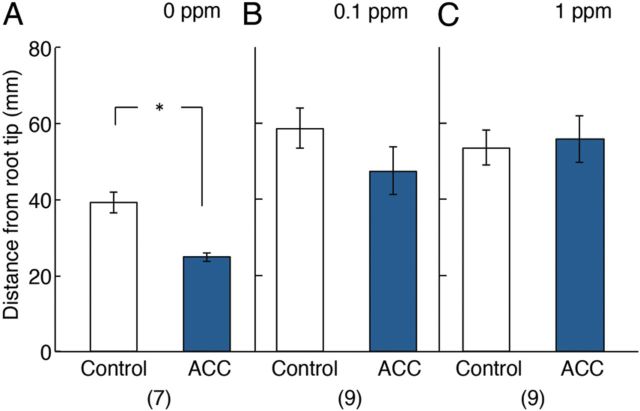
Effects of 4 days unilateral treatment of primary roots of rice with 1 µM ACC on the distance from root tip to where percentage of cross-sectional area of aerenchyma reached 40 % of the area of the cortex. Measurements were made in the presence or absence of 1-MCP. (A), (B) and (C) correspond to the treatment with ACC in the presence of 0, 0.1 and 1 ppm of 1-MCP, respectively. White column, control side; blue column, 1 μM ACC treatment side. Values are means ± SE. *P < 0.05 (Wilcoxon signed-ranks test). The numbers of samples are shown in parentheses.

Statistical analyses of the differences in the distance from the root tip to where aerenchyma development reached 40 % between each side are shown in Fig. 7. They indicate that aerenchyma developed significantly earlier in the ACC-treated side in the unilaterally treated roots in the absence of 1-MCP (Fig. 7A). On the other hand, in roots unilaterally treated with ACC in the presence of 1-MCP at 0.1 or 1 ppm, the bringing forward in time (based on position) of aerenchyma formation was cancelled (Fig. 7B and C). At the higher concentration of 1-MCP, aerenchyma formation in the side not given ACC was also delayed by the inhibitor.

## Discussion

When percentage of the root cortex comprising aerenchyma area was compared on a cross-section taken 50 mm from the root tip, the percentage significantly increased in the ACC-treated side (Fig. 4A). The fact that the comparison between the ACC treatment with the control was performed at the same distance (50 mm) from the root tip in one root means that this comparison was made at the same cell age. Furthermore, estimated total aerenchyma volume along the whole sides of the root significantly increased in the ACC-treated side (Fig. 6A). These results indicated that the treatment with ACC promoted aerenchyma development on a statistical basis.

In addition, the distance from the root tip where aerenchyma development reached 40 % of the cross-sectional area of the cortex significantly decreased in the ACC-treated side (Fig. 7A), indicating that ACC promoted aerenchyma development by accelerating its development compared with similar aged tissue on the opposite side of the root. These significant promotive effects of ACC were, however, largely overcome by supplying 1-MCP at 0.1 or 1 ppm (Figs. 4B, C, 6B, C, 7B and C). Together, these results indicated the ability of ethylene applied as ACC to promote aerenchyma development in rice roots in terms of amount and speed of formation. This, in turn, indicates that the extent of cell death is increased and the time necessary for the process of cell death is shortened.

Because no significant difference was found between the length of roots unilaterally treated with ACC in the presence and absence of 0.1 ppm 1-MCP (Fig. 2A and B) (Mann–Whitney *U* test, two-tailed, *P* > 0.05), the effects of 0.1 ppm 1-MCP on aerenchyma formation in the control sides were compared with regard to the following aspects: (i) the percentage of aerenchyma area (Fig. 4A and B, Control), (ii) the estimated volume of aerenchyma (Fig. 6A and B, Control) and (iii) the distance from the root tip to where aerenchyma development reached 40 % (Fig. 7A and B, Control) of roots unilaterally treated with 1 µM ACC. As a result, slight but significant differences were found in the effects of 0.1 ppm 1-MCP with regard to (i) and (iii) (Student's *t*-test, two-tailed, *P* < 0.05). Because the length of roots unilaterally treated with ACC was longer in the presence of 1 ppm 1-MCP than in its absence (Fig. 2A and C) (Mann–Whitney *U* test, two-tailed, *P* < 0.01), it was not possible to compare the effects of 1 ppm 1-MCP on aerenchyma formation since cells at the same position along the root would have different ages. However, the inhibiting effect of 1 ppm 1-MCP on the total volume of aerenchyma along the whole length of the control side root is not confounded by such age differences. The results here show a slight inhibition of constitutive aerenchyma by 1 ppm 1-MCP along the control side of the root (Fig. 6C) and on the distance from root tip where the percentage of cross-sectional area of aerenchyma reached 40 % of the area of the cortex (Fig. 7C). Overall, these results show (i) a promoting effect of exogenous ethylene (given as ACC) on aerenchyma development in the treated side of the root and (ii) the involvement of endogenous ethylene in promoting constitutive aerenchyma in the control side. Interestingly, root elongation was slowed in control roots at the higher 1-MCP concentration of 1 ppm (Fig. 2C). This result indicates that naturally occurring levels of ethylene in control roots stimulate root elongation, which is in line with previous studies (Smith and Robertson 1971; Konings and Jackson 1979).

In the present work, our ‘sandwich’ method is shown to be a useful method to detect effects of an environmental factor on root development. Here we show that the method is sufficiently sensitive to detect a promotive effect of ACC on aerenchyma formation and its cancellation by the ethylene action inhibitor 1-MCP. This indicates the potential for ethylene to promote aerenchyma development in rice roots. Furthermore, the technique also showed that aerenchyma development was slowed by 1-MCP even in the absence of added ACC. This indicates that the tendency for rice roots to form aerenchyma constitutively and without external stimulation from environmental stress such as oxygen shortage is also under ethylene regulation. The sandwich technique also has the potential to address the signalling mechanism involved in aerenchyma formation, including, for example, studying the activity of free reactive oxygen species (Nishiuchi *et al.* 2012; Yamauchi *et al.* 2013) and assessing their responsiveness and sensitivity of roots to hormones, inhibitors and environmental factors. Such work should aid our understanding of the physiological basis of stress tolerance in crop plants including flooding tolerance.

## Sources of Funding

Our work was supported in part by JSPS KAKENHI Grant (No. 24620003) to I.K.

## Contributions by the Authors

K.Y. performed research. K.Y. and I.K. analysed data. I.K. designed research and wrote the paper.

## Conflicts of Interest Statement

None declared.
